# Knowledge, attitude and use of herbal medicine among pregnant women attending antenatal care in nsukka, Nigeria

**DOI:** 10.4314/ahs.v24i4.24

**Published:** 2024-12

**Authors:** Obinna Felix Dim, Chigozie Gloria Anene-Okeke, Chinwe Victoria Ukwe

**Affiliations:** Department of Clinical Pharmacy and Pharmacy Management, Faculty of Pharma- ceutical Sciences, University of Nigeria, Nsukka, PMB 41001 Enugu State, Nigeria

**Keywords:** Knowledge, attitude, use, herbal medicine, pregnant women

## Abstract

**Objective:**

To assess the knowledge, attitude and use of herbal medicine among pregnant women attending antenatal in Nsukka, Enugu state Nigeria.

**Methods:**

This was a cross- sectional descriptive study carried out among pregnant women attending ante- natal in selected hospitals in Nsukka, Enugu State from June-August 2022. A 33 item well-structured questionnaire was used as a data collection tool. Data were analyzed using the IBM Statistical Product for Services Solution (SPSS) for Windows, Version 27.0. Descriptive and inferential statistics were utilized. Statistical significance was set at P< 0.05.

**Results:**

Out of 400 consenting pregnant women attending antenatal, above half were between the ages of 25 to 34, 233(58.3%), and are married 371(92.8%) with education within the tertiary category 193(48.2%). Above half had good knowledge 264(66.2%) of herbal medicine. Majority had positive attitude towards the use of herbal medicine 284(71.2%). More than half 226(56.5%) of the pregnant women had used herbal medicine apart from during pregnancy. Only few pregnant women 64(16%) had used herbal medicine in previous pregnancy.

**Conclusion:**

Majority of pregnant women that participated in the study had good knowledge and positive attitude towards herbal medicine.

## Introduction

Herbal medicines and natural health products are increasingly drawing the attention of regulators, researchers, and health professionals due to high levels of consumption worldwide.^1^ The desire to capture the wisdom of traditional healing systems has led to a resurgence of interest in herbal medicines,^2^ which lead to its practice in countries where conventional medicines are predominant.^3^ Herbs in this study involve the use of plant products in their raw or cooked forms which have not been subjected to laboratory investigations for their safety and efficacy.^4^ The World Health Organization reported that 70% to 80% of the world population relies mainly on herbal sources for their primary medicines.^3^

The use of herbal medicine in Nigeria is usually perceived as a rural practice, but the knowledge and use of herbal medicine in the urban areas of the country can also be observed. Several factors can contribute to the increasing awareness with the use of herbal medicine in developed and developing countries.^5^ Holistic approach to health problems and safety have been the particular influence of herbal medicine use to developed countries whereas their accessibility, affordability, historical, cultural, and religious backgrounds in addition to the above factors influence herbal medicine use in developing countries like Nigeria.^4^,^5^ In developing countries like Philippines, women, about 60% are using herbal remedies to alleviate some physiologic changes that occur during pregnancy.^6^ It is known that about 65 to 80 % of the world's population use herbal medicines as their primary form of health care. Studies have also reported different characteristics of women, which makes them more likely to take herbal medicine in pregnancy. These included being older, married, primiparous, being less educated and severity of nausea and vomiting etc.^7^

Many studies have been published on medications used during pregnancy, however there is still knowledge gap among pregnant women particularly in rural areas in Africa,^8^ since many women used herbal medicines during pregnancy, which might produce potential adverse side effects on the mother and fetus, such practice modalities should raise concerns among healthcare professionals and consumers on the issue of safety and efficacy. There is however, paucity of information on the general knowledge, attitude and use of herbal medicine among pregnant women attending antenatal care in Nigeria and since many women use herbal medicines during pregnancy, which might produce potential adverse side effects on the mother and fetus, it is necessary to determine the level of knowledge, attitude and use of these herbal medicines among pregnant women attending antenatal in Nsukka, Nigeria.

## Methods Study Design

This was a cross- sectional descriptive study carried out to assess the knowledge, attitude and use of herbal medicine among pregnant women in Nsukka, Enugu State, Nigeria.

### Study Settings

The study was carried out in Nsukka, Enugu State, Nigeria. Nsukka is located about 72.8km from Enugu city, which is the capital of Enugu state Nigeria. Nsukka also shares common border with Enugu Ezike and obollo-Afor, Ede- Oballa, Uzo Uwani and mkpologwu, all which are in Nsukka senatorial zone. The total population of Nsukka at the 2006 census was 309,633. Nsukka is the second town in Enugu state.

The hospitals used for the study were Bishop Shanahan memorial hospital and faith foundation hospital, Nsukka. These settings were selected because they are the major hospitals in the locality and due to the vast use of herbal medicines in general treatment of diseases.

The study population were pregnant women attending ante-natal clinic in some selected hospitals in Nsukka town from June-August 2022 and met inclusion criteria. These hospitals were conveniently selected because of the number of pregnant women that receive ante-natal care from these hospitals.

### Sample Size

All pregnant women that received antenatal care within the period of the study and are 18 years and above, were utilized for the study.

### Sampling Techniques

The patients were conveniently samples based on pregnant women that were available for antenatal care on the respective days when data was collected.

### Eligibility criteria

Pregnant women who were aged 18-60 years, registered for antenatal care in the various hospitals, gave oral consent and agreed to participate in the study.

### Instrument for data collection

The study employed a well-structured questionnaire, used as the data collection tool. The questionnaire is a 33 item –question, which comprise of thirty-one closed-ended questions and two opened-ended questions. The questionnaire is divided into four sections; section A- socio- demographic characteristics of the participants, it has about nine demographics which include the age of the participants, marital status, educational status, occupation, monthly income, place of residence, no of children, and history of hospitalization after taking herbal medicine. section B; knowledge of herbal medicine among pregnant women, comprises of ten knowledge of use questions with a dichotomous (yes/no) response format. Yes, was coded as ‘0’ while No was coded as ‘1’. Section C; attitude of herbal medicine among pregnant women comprises of a 12 item questions which has a 5- point likert scale ranging from Strongly disagreed (SD), Disagreed(D), neutral(N), agreed(A) and strongly agreed (SA). SD, D, N, A, SA were all coded as 0,1,2,3,4 respectively. Section D; use of herbal medicine among pregnant women. This section comprised of a nine-item question on herbal medicine use in Nsukka. The questions are mostly dichotomous (Yes/No), however some of the questions are self-reported. Questions such as Herbal medicine are plant based. Herbal medications generally do not have dosage instruction therefore unsafe for use in pregnant women. Yoyo bitter which is an example of herbal medicine, when taken in excess can cause both liver and kidney failure, were asked to the patient to ascertain their knowledge level on herbal medicine use. Also, questions such as Herbal medicine has fewer side effects than conventional medicine during pregnancy, Herbal medicine is more accessible than conventional medicine. Were used to access the attitudes of pregnant woman towards herbal medicine use.

### Data collection

The instrument for data collection was paper based. Some questions were extracted from previous studies on knowledge, attitude and Use of herbal medicines in other countries.^1^,^6^,^13^. Participants were briefed on the importance of the study. Questions that appeared ambiguous was carefully explained to the participants. The questionnaire was self-administered to 400 consenting pregnant women attending ante-natal care. The pregnant women were asked to fill the questionnaire immediately when given and it was retrieved immediately after completion to avoid sourcing information on the internet, since the research is knowledge based. Confidentiality was maintained throughout the study period.

### Data Analysis

Data was coded and entered into Microsoft-Excel, then exported, cleaned and analyzed using the IBM Statistical Products and Service Solutions (SPSS) for Windows, Version 27.0 (IBM Corp, Version 27.0, Armonk, NY, USA). Descriptive statistics such as frequency and percentages were used to describe socio-demographic characteristics of participants. The mean of the knowledge and attitude were determined and used to determine pregnant women with good or poor knowledge and those that have positive or negative attitude towards the use of herbal medicine in Nsukka, Nigeria.

### Ethical consideration

Ethical approval for this study was granted by the Health Research Ethics Committee of University of Nigeria Teaching Hospital, Enugu state, before the study commenced- NHREC/05/01/2008B-FWA00002458-1RB00002323. Informed consent was obtained from each respondent.

## Results

A total of 450 questionnaires were distributed to about 450 consenting mothers that reported for ante natal care however only about 400 questionnaires were correctly filled and returned giving a response rate of 88.8%. Table 1 depicts the sociodemographic characteristics of pregnant women receiving prenatal treatment in Nsukka. According to the table, the majority of the women included in the study were between the ages of 25 and 34 (233, 58.3%), were married (371, 92.8%), and had higher education (193, 48.2%). The majority of women seeking prenatal treatment in Nsukkawerecivilservants 167 (41.7%). The majority of the women live in the town's urban area and have one to four children 268 (67%).

**Table 1a:** Socio-demographic characteristics of respondents

Variables	N	(%)
**Age of respondents**		
18-24	132	33.0
25-34	233	58.3
35-44	30	7.5
45-54	5	1.2
**Marital status**		
Single	8	2.0
Married	371	92.8
Divorced	10	2.5
Widowed	11	2.7
**Educational status**		
No formal education	12	3.0
Primary	51	12.8
Secondary	144	36.0
Tertiary	193	48.2
**Occupation**		
Unemployed	71	17.8
Civil servant	167	41.7
Self –employed	162	40.5
**Monthly income (in Naria)**		
<18,000	111	27.8
18,000-50,000	195	48.7
50,000-100,000	60	15
>100,000	34	8.5

**Table 1b:** Socio-demographic characteristics of respondents

Variable	N	(%)
**Income description**		
Not sufficient	187	46.7
Meets the need	161	40.3
Allow savings	52	13
**Place of residence**		
Nsukka Village	135	33.7
Nsukka town	251	62.8
Not living in Nsukka	14	3.5
**Number of children**		
No child	60	15
1-4 children	268	67
>4 children	72	18
**History of hospitalization after taking herbal medicine**		
No	314	78.5
Yes	86	21.5

N=400

Table 2 shows the knowledge of pregnant women on herbal medicine. In order to determine the knowledge of herbal medicine among pregnant women, statements such as 1. “Herbal medications generally do not have dosage instructions therefore unsafe for use in pregnant women”, 2. “herbal medicine are plant-based substances and the use of yoyo bitter can be harmful to my fetus”, generated the highest number of correct responses 281(70.3%);388(97%);330(82.5%) respectively. The mean knowledge score of herbal medicine among pregnant women was 66.89±14.63, Above half had good knowledge 264(66.2%) of herbal medicine.

**Table 2:** Knowledge of herbal medicine among pregnant women

Variables	n(%)	

	Incorrect	Correct
Herbal medicine are plant based substances	12(3.0)	388(97.0)
The use of Yoyo bitter can be harmful to my foetus	70(17.5)	330(82.5)
Garlic can lower my blood lipid level during pregnancy	152(38.0)	248(62.0)
Ginseng can be used safely in pregnant mothers with high blood pressure	165(41.3)	235(58.7)
Uziza could be used to stop nausea and help set the stomach well after delivery	137(34.3)	263(65.7)
Dogoyaro (Neem plant) can be used to cure malaria in pregnancy	220(55.0)	180(45.0)
Herbal medications generally do not have dosage instructions therefore unsafe for use in pregnant women	119(29.7)	281(70.3)
Ginseng should be avoided in pregnant diabetic patients	150(37.5)	250(62.5)
Use of kola nut is recommended during my pregnancy	249(62.3)	151(37.7)
Yoyo bitter when taken in excess can cause both liver and kidney failure	52(13.0)	348(87.0)

N=400

Table 3, shows the attitude of pregnant women towards herbal medicine. When the women were asked if herbal medicine was more effective than conventional medicine, majority of the women strongly agreed to that assertion 174(43.5%) with a Mean± SD 3.85± 1.29. when women were asked if herbal medicine has fewer side effects than conventional medicine, one - thirds of the population strongly agreed. Most women strongly agreed that in some conditions, herbal medicine effects are faster in action than conventional medicine. Overall, Majority had positive attitude towards the use of herbal medicine 284(71.2%)

**Table 3:** Attitude of pregnant women toward herbal medicine

Variable	Strongly disagreed	Disagreed	Neutral	Agreed	Strongly Agreed	Mean± SD
Herbal medicine are more effective than conventional medicine	27(6.8)	51(12.8)	53(13.3)	95(23.8)	174(43.5)	3.85±1.29
Herbal medicine are more effective than conventional medicine for some medical condition	62(15.5)	108(27.0)	29(7.3)	110(27.5)	91(22.8)	3.15± 1.43
Herbal medicine when combine with conventional medicine are effective	32(8.0)	39(9.8)	59(14.8)	123(30.8)	147(36.8)	3.79±1.26
Herbal medicine has less side effects than conventional medicine	32(8.0)	34(8.5)	71(17.8)	159(39.8)	104(26.0)	3.67±1.18
Herbal medicine has less side effects than conventional medicine during pregnancy	21(5.3)	51(12.8)	54(13.5)	128(32.0)	146(36.5)	3.82±1.20
Herbal medicine is more accessible than conventional medicine	24(6.0)	55(13.8)	22(5.7)	131(32.8)	166(41.5)	3.90±1.25
Herbal medicine is less expensive than conventional medicine	21(5.3)	30(7.5)	13(3.3)	148(37.0)	184(46.0)	4.12±1.13
Herbal medicine is accessible without doctor's prescription	29(7.3)	26(6.5)	15(3.8)	157(39.3)	170(42.5)	4.04±1.18
In some condition, Herbal medicine effects are faster than conventional medicine	91(22.8)	94(23.5)	50(12.5)	72(18.0)	93(23.3)	2.96±1.50
In all I prefer to use herbal medicine for some condition such as malaria during pregnancy	29(7.3)	44(11.0)	46(11.5)	155(38.8)	126(31.0)	3.76±1.21
If conventional medicine don't have effect, I would try herbal medicines	116(29.0)	53(13.3)	62(15.5)	95(23.8)	70(17.5)	2.87± 1.50
If my caregiver offers, I would try herbal medicine	130(32.5)	65(16.3)	44(11.0)	100(25.0)	58(14.5)	2.73±1.49

Out of the twelve questions, the question ‘Herbal medicine is less expensive than conventional” medicine had the highest response (N=184) of strongly agreed (46.0%) with a mean and standard deviation of 4.12±1.13, followed by ‘Herbal medicine is accessible without doctor's prescription’ with the response (N=170) of strongly agreed (42.5%) with a mean and standard deviation of 4.04±1.18.

More than half (56.5%) of the pregnant women had used herbal medicine apart from during pregnant. Only few pregnant women (16%) had used herbal medicine in previous pregnant and less than half (40%) of the pregnant women were using herbal medicine for their current pregnancy as seen in table 4a and 4b respectively.

**Table 4a:** Use of herbal medicine in pregnancy

Questions	Yes	No
Apart from during pregnancy, have you ever used herbal medicine	226(56.5)	17(43.5)
Have you ever used herbal medicine in previous pregnancy	64(16)	336(84.0)
Have you ever used herbal medicine during your current pregnancy	160(40)	240(60)
	**Frequency**	**Percentage**
If yes, which period of pregnancy		
First trimesters	120	30.0
Second trimester	27	6.8
Third trimester	33	8.2
No response	220	55.0
Herbal medicine use dangerous in?		
First trimesters	216	54.0
Second trimester	32	8.0
Third trimester	32	8.0
Throughout pregnancy	73	18.2
No response	47	11.8

N=400

**Table 4b:** Use of herbal medicine in pregnancy

Variable	Frequency	Percentage
Why are you using herbal medicine?		
It is our traditional medicine	102	25.5
I have been using it from birth	37	9.2
It is cheap	146	36.5
I don't know	104	26.0
No response	11	2.8
How do you classify/ consider the product used?		
Herbal remedied/product	82	20.5
Food supplement	81	20.3
Phytomedicine	30	7.5
Homeopathic medicine	25	6.2
Natural product	108	27.0
Any other way	74	18.5

Table 5 depicts the relationship between Sociodemographic Characteristics and Knowledge of use of herbal medicine among pregnant women in Nsukka. Of all the sociodemographic variables, only marital status was statistically significant.

**Table 5a:** Relation ship between Sociodemographic Characteristics and Knowledge Level of Pregnant Women

Characteristics	Wrong(%)	Correct(%)	*P Value*
**Age (Years)**			
18-24	39.5	28.3	0.133
25-34	52.1	62.7	
35-44	7.2	7.7	
45-54	1.2	1.3	
**Marital Status**			0.012*
Single	3.6	0.9	
Married	94.0	91.8	
Divorced	0.0	4.3	
widowed	2.4	3.0	
**Educational Status**			0.325
No formal education	1.2	4.3	
Primary	12.0	13.3	
Secondary	37.1	35.2	
Tertiary	49.7	47.2	
**Occupation**			0.503
Unemployed	16.1	18.9	
Civil services	41.0	42.5	
Self-employed	42.9	38.6	

**Table 5b:** Relationship between Sociodemographic Characteristics and Knowledge Level of pregnant women

Characteristics	Wrong(%)	Correct(%)	*P Value*
**Monthly income**			0.484
<18,000	28.2	27.0	
18,000-50,000	51.0	47.6	
50,000-100,000	12.0	17.2	
>100,000	8.8	8.2	
**Income description**			0.194
Not sufficient	51.8	43.1	
Meets the need	37.5	42.2	
Allows savings	10.7	14.7	
**Number of children**			0.551
No child	17.0		
1-4	65.5	67.8	
>4 children	17.5	18.5	
**History of hospitalization**			0.052*
Yes	73.8	81.9	
No	26.2	18.1	

## Discussion

The study offers insights into the knowledge, attitude, and use of herbal medicine among pregnant women attending antenatal in Nsukka. From the results obtained, pregnant women in Nsukka had good knowledge and positive attitude toward herbal medicine. The socio-demographic characteristics reveal that a majority of the respondents were aged 25-34, married, and had attained tertiary education. Similar study conducted by fakaye et al reported a somewhat similar age range of 21-30 year.^10^ Also, a study by Olowokere et al reported 16-30 years. This demographic profile is crucial for understanding the factors that may influence knowledge, attitudes, and practices related to herbal medicine use during pregnancy^11^.

The findings indicate a good level of knowledge regarding the plant-based nature of herbal medicines. However, misconceptions exist, with a substantial proportion believing that Yoyo bitter cannot be harmful to the fetus and that garlic can lower blood lipid levels during pregnancy. These results underscore the importance of targeted educational interventions to address misconceptions and promote accurate information about herbal medicine during pregnancy

Knowledge score of herbal medicine among pregnant women contradicts with the findings of the study carried out in Southern Ethiopia, where they reported higher knowledge (80.7%) than from this study.^7^ Another study carried out in Malaysia to assess Women's Knowledge and Practice of herbal medicine use, reported low knowledge score among the women.^9^ The differences may be due to the numbers of respondents and country where the research was conducted. Majority of the respondents had good knowledge that herbal medicines are plant-based substances this correlated with these studies.^12^,^13^,^14^,^15^

Several studies have reported variation in demographic characteristics of women that more likely to use herbal in pregnancy. These demographics variation include being older in age, married, having tertiary education, being less educated and primiparous.^11^,^12^ This finding is in contrast to a study carried out among Malaysian women where only income was associated with knowledge score (< 0.05)^9^.

Attitudes are learnt over time and are influenced mainly by culture and beliefs of the people and are very difficult to change.^13^ Most of the Africa cultural beliefs strongly in herbal medicine use and its efficacy over centuries and are passed from generations to generations thus affecting our attitudes and behaviours towards herbal.^16^ The findings of our study showed that the pregnant women had positive attitude toward herbal use which is in line with the findings of a study carried out by laelago et al that 52.1% of the pregnant women had positive attitude toward herbal medicine.^7^ Another study carried out in eastern Nigeria showed negative attitude toward herbal medicine^16^.

Less than half of pregnant women in this study used at least one herbal medicine during their current pregnancy. This prevalence is higher than the 12.1% and 31.4% reported by a study conducted in Western and Northern Nigeria.^17^,^18^ The use of herbal medicine may probably be due to the strong believes that these herbs are safe since they are plant based derived substances. Studies have reported that most women believe in the safety of herbal medicine use in pregnancy and the perceived absence of side effects.^16^-^18^ Less than half of the respondent used herbal medicine in the first trimester of their current pregnancy. This is in line with other studies which reported the use of herbs during the first trimester; probably due to the higher incidence of pregnancy-related problems during this period such as nausea, vomiting, weakness etc.^19^ Although Sooi et al had a contrary opinion that the highest use of herbal medicine in pregnancy is during labour.^9^

The study reveals a considerable herbal medicine use among pregnant women, with a notable concentration during the first trimester. This aligns with existing literature^7^,^16^,^18^ highlighting a common reliance on traditional remedies during the early stages of pregnancy. Interestingly, the perceived danger associated with herbal medicine use, particularly in the first trimester, suggests a level of knowledge among participants regarding potential risks. This knowledge underscores the need for healthcare providers to acknowledge and address concerns related to safety during specific pregnancy periods.

The reasons for herbal medicine use vary, with tradition and cost-effectiveness emerging as significant factors. The diverse classification of herbal products, ranging from natural products to herbal remedies, signals a need for standardization and clear communication about these products. This diversity may complicate healthcare providers' efforts to guide pregnant women effectively. Studies however revealed that pregnant women use herbal medicine for numerous reasons such as safety, natural, less side effect, affordability, labour facilitation.^10^,^11^,^12^,^13^,^14^

## Study Limitations

The study, while providing valuable insights into the knowledge, attitudes, and practices of pregnant women Regarding herbal medicine in Nsukka, has several limitations that warrant consideration. Firstly, the findings may not be broadly applicable due to the focus on a specific geographical location, Nsukka. Therefore, caution should be exercised when generalizing the results to other regions or diverse populations. Secondly, the study's cross-sectional design limits its ability to establish causal relationships. Since it provides a snapshot of participants' perspectives at a particular moment, it's challenging to discern temporal trends or changes in attitudes and practices over time. Thirdly, the reliance on self-reported data introduces the possibility of recall bias and social desirability bias. Respondents may alter their answers based on what they think is socially acceptable, potentially leading to inaccuracies in reported behaviors. Lastly, the study's scope regarding the reasons for herbal medicine use during pregnancy is limited. While it acknowledges this practice, it does not delve deeply into the specific motivations behind it. Acknowledging these limitations provides a context for interpreting the study's findings and underscores the need for further research to address these constraints and contribute to a more comprehensive understanding of the subject matter.

## Conclusion

This study showed a considerable use of herbal medicine among pregnant women. It provides valuable insights into the multifaceted landscape of herbal medicine knowledge and attitude among pregnant women in Nsukka. The pregnant women had good knowledge and positive attitude towards herbal medicine. The identified knowledge gaps and misconceptions emphasize the critical role of targeted education campaigns. The positive attitudes toward herbal medicine underscores the importance of open and non-judgmental discussions between healthcare providers and pregnant women. Standardizing communication about herbal products is pivotal to ensure that accurate information is disseminated, promoting the safe and effective use of herbal remedies during pregnancy.

## Recommendations

Given the observed good knowledge, positive attitudes and use of herbal medicine among pregnant women in Nsukka, there is a pressing need for healthcare policymakers, practitioners, and educators to integrate this awareness into existing maternal health programs. Incorporating information on herbal medicine use during pregnancy into antenatal care programs can empower healthcare providers to offer more comprehensive and culturally sensitive guidance. This study serves as a valuable resource for shaping maternal healthcare policies, interventions, and educational programs in Nsukka, Enugu State, and beyond.
